# Relationship of phytochemicals and antioxidant activities in *Gymnema inodorum* leaf extracts

**DOI:** 10.1016/j.heliyon.2023.e23175

**Published:** 2023-12-02

**Authors:** Onanong Nuchuchua, Wanwisa Srinuanchai, Chaisak Chansriniyom, Uthaiwan Suttisansanee, Piya Temviriyanukul, Nitra Nuengchamnong, Uracha Ruktanonchai

**Affiliations:** a National Nanotechnology Center (NANOTEC), National Science and Technology Development Agency (NSTDA), Pathum Thani, Thailand; b Department of Pharmacognosy and Pharmaceutical Botany, Faculty of Pharmaceutical Sciences, Chulalongkorn University, Bangkok, Thailand; c Natural products and Nanoparticles Research Unit, Chulalongkorn University, Bangkok, Thailand; d Institute of Nutrition, Mahidol University, Nakhon Pathom, Thailand; e Science Laboratory Center, Faculty of Science, Naresuan University, Phitsanulok, Thailand; f National Science and Technology Development Agency (NSTDA), Pathum Thani, Thailand

**Keywords:** *Gymnema inodorum* leaf extract, Phenolics, Flavonoids, Mass spectrometry, Antioxidations, Vascular protection

## Abstract

*Gynmena inodorum* (GI) is a green leafy vegetable used in the Northern Thai cuisine which has antioxidant activities and may be applicable for preventing oxidative stress and aging-related disease. However, understanding the relationship between GI phytonutrients and their antioxidant properties has been unclear. The aims of this study were to identify the GI leaf phytochemicals and to study their antioxidant activities. A chromatogram of LC-ESI-MS/QTOF-MS showed that the GI leaves were potentially composed of phenolics, quinic acids, flavonoids, and triterpenoid saponins. This study was able to authenticate quercetin, kaempferol, and triterpenoid GIA1 in the samples. The GI materials with high contents of phenolics, flavonoids, quercetin, and kaempferol showed significant relation to antioxidation and protection in endothelial cell death suppressed by reactive nitrogen species. Meanwhile, triterpenoids had a low antioxidant impact. Ultimately, GI leaves with high phenolic compounds are a promising raw material to develop as an antioxidant functional food.

## 1 Introduction

Fruits and vegetables are dietary sources of phytonutrients including simple phenols, phenolic acids, flavonoids, coumarins, stilbenes, tannins, lignans, and lignins. These compounds are naturally synthesized from primary metabolites (carbohydrates, lipids, and amino acids) and used to protect against UV radiation, insects, viruses, and bacterial infections [[1], [2], [3]]. Recently, plant phenolics have been acknowledged as strong natural antioxidant agents and are considered a vital human dietary component. An intake of dietary phenolics either alone or in combination is recommended to reduce the risk of many oxidative stress-related diseases such as cancers, diabetes, and cardiovascular disease [4,5].

Type 2 diabetes mellitus (T2DM) is an example of non-communicable diseases that can associate with a rise in oxidative agents due to an abnormal glycolysis pathway [6]. High blood sugar levels for a long period of time in diabetic patients can induce production of oxidative agents by an auto-oxidation of glucose [7], including oxygen-reactive species [8] and nitrogen reactive species [9,10]. Long-term oxidative stress accumulation further affects macro- and microvascular dysfunction, resulting in diabetic complications [11,12]. Diabetes management mostly focuses on controlling hyperglycemia by using therapeutic agents, diet controls, and lifestyle changes [[12], [13], [14]]. Meanwhile, a reduction of intracellular free radical formations would provide a therapeutic strategy to prevent and restore cell damages from oxidative stress. Thus, the consumption of healthy diets, such as fruits, vegetables, and natural products containing large groups of biologically active substances, is anticipated to promote co-benefits of anti-diabetes and antioxidation.

*Gymnema inodorum* (GI), or Chiang-Da, is a traditional culinary and medicinal plant in the northern region of Thailand. Its young leaves are used as a raw cooking and dietary supplement industry material. *G. inodorum* has shown anti-diabetes, such as glycemic controls by inhibiting α-glucosidase and α-amylase activities [15,16] and reducing intestinal glucose absorptions [15,17], anti-inflammation [[18], [19], [20]], anti-insulin resistant and anti-insulin mimetic activities by increasing glucose uptake into adipocytes without insulin signaling [20], and anti-adipocyte differentiation [21]. Other health-promoting effects include antioxidant properties [19,22], anti-malarial potential and protection from malaria-associated cardiac injury [23,24].

*G. inodorum* leaf (GIL) phytochemicals are composed of triterpene saponins [15,17,25], pregnane glycosides [16], phenolic compounds [26], vitamins, carotenes and xanthophylls [22]. Saponin derivatives and pregnane glycosides are directly associated with anti-diabetic controls [15,16,25]. The antioxidant not yet activities of GI leaves were also reported, but their related phytochemicals have been identified. The present study investigates and seeks a better understanding of the relationship between phyto-compositions and the antioxidant impacts of GI leaves.

This research aimed to screen GIL phytochemicals by liquid chromatography-electrospray ionization quadrupole time-of-flight mass spectrometry (LC-ESI-MS/QTOF-MS). The GI leaf samples were randomly collected from five different areas. The samples of GIL phytochemicals were prepared by ethanolic maceration following the method detailed in our previous study [15]. The different antioxidant mechanisms were conducted following DPPH, ABTS, FRAP and ORAC methods. Cellular antioxidation was also observed on HUVEC damages by reactive nitrogen species. Moreover, Pearson's correlation, principal component analysis (PCA), and hierarchical cluster analysis (HCA) were used to analyze the phytochemicals related to antioxidant activities.

## 2 Materials and methods

### 2.1 Preparation of *G. inodorum* extracts

*G. inodorum* leaf (GIL) samples were obtained from different areas as shown in Table S1. The preparation of the GIL extracts was carried out following the protocol of Srinuanchai et al. [15]. Briefly, 100 g of the dry leaf sample was ground and subsequently soaked with 1000 mL of 75%v/v ethanol. The extractions were repeated in triplication. Then, the extract solutions were pooled, and filtrated through a filter paper. Ethanol residual was removed by a rotary evaporator. The crude extracts were dried by lyophilization and kept at −20 °C for further analysis.

### 2.2 Total contents of phenolics, flavonoids, and triterpenoids

The GIL extracts' total phenolic contents (TPCs) were determined by Folin-Ciocalteu assay following the method of Park and Lee [27] with minor modification. Briefly, the samples were prepared at 200 μg/mL in 50 % ethanol. Next, the sample solutions (50 μL) were mixed with 125 μL of 10 % Folin-Ciocalteu reagent and incubated for 3 min at room temperature. Then, 2 % Na_2_CO_3_ (750 μL) was added to the mixtures and incubated for 30 min in the dark. The absorbance of the sample solutions was measured at 765 nm using a spectrophotometer (VICTOR Nivo, PerkinElmer Inc., Shelton USA). TPCs were calculated using a standard curve of gallic acid (0−50 μg/mL). The TPCs in the GI extracts are shown in milligrams of gallic acid equivalents per 1 g of crude extract (mg GAE/g extract).

The GIL extracts' total flavonoid contents (TFCs) were determined using a modified aluminum chloride colorimetric method [28]. The extract solutions were prepared at 100 μg/mL. Then, 175 μL of the sample solution was mixed with 75 μL of 5 % sodium nitrite. The mixtures were incubated at room temperature for 6 min. Later, 2 % aluminum chloride (125 μL) was added and further incubated for 6 min. Then, 1 M sodium hydroxide (125 μL) was added and left in the dark at room temperature for 10 min. The absorbance of the reaction mixtures was measured at 415 nm with a spectrophotometer. The calculation of TFCs was compared to 0−50 μg/mL of a standard quercetin. The TFCs in the extracts are shown in milligrams of quercetin equivalents per 1 g of crude extract (mg QE/g extract).

The GIL extracts ' total triterpenoid contents (TFCs) were determined according to Wang et al. [29]. Briefly, 100 μL of the extract solutions (1000 μg/mL) was dried in a vacuum oven overnight. After that, 150 μL of 5%w/v vanillin in acetic acid and 500 μL of perchloric acid were added to the dry samples. The mixtures were incubated at 60 °C for 45 min after adding 2.25 mL of acetic acid. The color intensity of the reaction mixtures was measured at 548 nm. TTPs of the extracts were compared to ursolic acid (0−300 μg/mL). The results are shown in milligrams of ursolic acid equivalents per 1 g of crude extract (mg UAE/g extract).

### 2.3 Phytochemical screening by liquid chromatography-electrospray ionization quadrupole time-of-flight mass spectrometry (LC-ESI-MS/QTOF-MS)

The chemical constituents contained in the extract were carried out following the work of Chansriniyom et al. [30]. Characterization was performed by Q-TOF mass spectrometer (Agilent 6540, Singapore) with a column ZORBAX Eclipse Plus C18 (4.6 mmx100 mm, 3**.**5 μm**)**. The mass spectrometric analysis was performed using an Agilent 6540 Q-TOF-MS spectrometer equipped with an electrospray ionization (ESI) source and Agilent dual nebulizer operating in a negative ionization mode. To identify phytonutrients, peak retention time, and mass data, their fragmented ions were compared to those of registered compounds on two databases, Human Metabolome Database (HMBD) and METLIN Metabolomics Database and Library (Agilent technology).

### 2.4 Quantification of GIA1 in GIL extracts

The determination of (3*β*, 16*β*)-16,28-dihydroxyolean-12-*en*-3-yl-*O*-*β*-*d*-glucopyranosyl-*β*-*d*-glucopyranosiduronic acid (GIA1) was carried out following the work of Srinuanchai et al. [15]. The chromatographic experiment was performed by Waters (Milford, MA, USA) equipped with an Alliance 2695 separation module and a 2998 photodiode array detector. The analyte was analyzed using Empower Pro 2 Software (Waters, USA).

Briefly, 20 μL of the standard GIA1 (0–100 μg/mL) and the extract samples (1000 μg/mL) were injected into a reverse phase column (Luna® C_18_ 100 Å 150 mm × 4.6 mm), connected to SecurityGuard™ C_18_ 4 mm × 2.0 mm from Phenomenex, Inc., Thailand. Separation was performed by two mobile phases: solution A (0.1%v/v formic acid in acetonitrile) and solution B (0.1 % v/v formic acid in DI water). A gradient elution was performed from 40%A to 75%A in 7 min. 75%A was held for 1 min before changing to 40%A in 1 min and held for 3 min. The total run time was 11 min. The flow rate of the system was 0.5 mL/min. GIA1 was detected by UV absorption at 210 nm.

### 2.5 Quantification of phenolic compounds in GIL extracts

The crude GIL extracts were pre-treated with acid hydrolysis following the protocol of Sirichai et al. [31]. The solvent was eliminated by rotary evaporation. Residues were reconstituted with methanol prior to analyzing by liquid chromatography-electrospray ionization tandem mass spectrometry (Dionex Ultimate 3000 series ultrahigh-performance liquid chromatography (UHPLC) system equipped with a diode array detector (DAD) from Thermo Fisher Scientific, Bremen, Germany). The ion fragmentation and mass analysis were operated by a TSQ Quantis Triple Quadrupole mass spectrometer (Thermo Fisher Scientific, Bremen, Germany) to obtain MS/MS ion fragments of authentic standards at specific collision energies and radio frequencies (RF−lens).

The analysis of phenolic compounds in the GI extracts was compared to authentic standards; phenolic acids (3,4-dihydroxybenzoic acid, chlorogenic acid, 4–hydroxybenzoic acid, caffeic acid, syringic acid, *p*-coumaric acid, ferulic acid, sinapic acid, cinnamic acid, gallic acid, vanillic acid, and rosmarinic acid); flavonoid standards ((−)-epigallocatechin gallate, hesperidin, myricetin, luteolin, quercetin, naringenin, kaempferol, apigenin, genistein, isorhamnetin, galangin, and rutin). The LC–ESI−MS/MS with a Chromeleon 7 chromatography data system (version 7.2.9.11323 from Thermo Fisher Scientific, Bremen, Germany) was used for molecular mass analysis of the phenolics. The ion fragmentations of standard chemicals were reported in the work of Sirichai et al. [31].

### 2.6 Antioxidant assays

*2,2-diphenyl-1-picrylhydrazyl (DPPH) radical-scavenging assay*: The extract solutions (100 μL) in ethanol at concentrations between 250 μg/mL and 850 μg/mL were mixed with 100 μL of 100 μM DPPH solution. A standard curve of a water-soluble vitamin E analogue (Trolox) was carried out at 1.5–6.0 μg/mL. The mixtures were incubated in the dark at 25 °C for 30 min and the color intensity was measured using a 96-well UV–visible microplate reader at 517 nm.

*2,2′-azino-bis (3-ethylbenzothiazoline-6-sulfonic acid) (ABTS•+) radical cation-based assays*: The extract solutions (20 μL) in ethanol at concentrations between 2500 μg/mL and 5500 μg/mL were mixed with 180 μL of the *ABTS•+* solution (14 mM ABTS and 4.9 mM potassium persulfate). A standard curve of Trolox was carried out at 40–100 μg/mL. The mixtures were incubated in dark at 25 °C for 30 min and the color intensity was measured using a 96-well UV–visible microplate reader at 750 nm.

The antioxidant activities of the extracts using DPPH and ABTS assays are presented in term of the Trolox equivalent antioxidant capacities (μmol TE/g extract) at 50 % of the radical scavenging activities.

*FRAP and ORAC methods*: The FRAP assay was detected using FRAP reagent at 595 nm. The FRAP activity was evaluated using the Trolox standard of 7.81–250.00 μM, while ORAC activity used the Trolox standard of 3.12–100.00 μM. The experiments followed the protocol of Sirichai et al. [31].

### 2.7 Screening of cellular antioxidant activity of the GIL extracts

Human umbilical vein endothelial cells (HUVEC, ATCC-CRL-1730™ from ATCC, VA, USA) were cultured in a F–12K medium containing supplementary components following a protocol of ATCC. After the cell confluency reached 80−90 %, HUVEC were seeded into a 96-well plate at the concentration of 50,000 cells/well. The GIL extracts at 0−62.5 μg/mL were treated on HUVEC for 24 h before incubating 14.5 μM peroxynitrite (Merck Ltd., Darmstadt, Germany) for 1 h. The cell viability of HUVEC was measured using the CellTiter 96® AQ_ueous_ One Solution Cell Proliferation Assay (Promega Corporation, Madison, WI, USA). When cell culture media with viable cells developed a purple color, the color intensity was determined by light absorption at 490 nm.

### 2.8 Data analysis

The relations of the phenolic compounds and antioxidations were analyzed using Pearson's correlation coefficient (*r*), Principal Component Analysis (PCA), and Hierarchical Cluster Analysis (HCA) by XLSTAT® (Addinsoft Inc., New York, USA). Moreover, a significant difference of data was performed at *p* values equivalent and less than 0.05, analyzed by One-way ANOVA using GraphPad Prism 8.0 (GraphPad software, Boston, MA, USA).

## 3 Results

### 3.1 Phytochemical screening by tandem mass spectrometry

Tentative phytonutrients of the *G. inodorum* leaf (GIL) were identified by tandem mass spectrometry (LC-ESI-MS/QTOF-MS). The GIL1 sample was randomly selected for this study. The ion chromatogram was extracted and shown in Fig. S1, Supplementary Material. The *m/z* data and their ion fragmentations (MS/MS) suggested the chemical structures of phenolics (P), quinic acid derivatives (QA), flavonoids especially quercetin glycosides (Q) and kaempferol glycosides (K), triterpenoids (T), amino acids, sugar alcohols and other unknown compounds (see Table 1).

**Table 1:** Tentative phytonutrients in *G. inodorum* leaf extract from a GIL1 sample, identified by LC-ESI-MS/QTOF-MS method using the negative ionization mode.

Peak no.	RT (min)	*m/z*	Adduct	MS/MS	Tentative identification	Formula	Error (ppm)
1	3.708	547.2016	[2 M + FA−H]^−^		*N*-(1-deoxy-1-fructosyl) alanine	C_9_H_17_NO_7_	4.00
1	3.892	181.0731	[M−H]^−^		mannitol	C_6_H_14_O_6_	−7.39
1	3.949	683.2302	[2 M−H]^−^		sucrose	C_12_H_22_O_11_	−7.40
1		477.1478	[M−H_20_–H]^−^		cappariloside B (**P1**)	C_22_H_28_N_2_O_11_	7.69
1	4.449	179.0564	[M−H]^−^		hexose	C_6_H_12_O_6_	−1.61
2	4.467	133.0147	[M−H]^−^		malic acid	C_4_H_6_O_5_	−3.41
3	5.987	290.0900		200.0539, 128.0332	unidentified		
4	6.188	372.1049		128.0338	unidentified		
5	7.261	257.0294		117.0173, 73.0281	unidentified		
5	7.340	115.0036	[M−H_2_O–H]^−^	73.0279	malic acid	C_4_H_6_O_5_	0.71
6	8.297	164.0725	[M−H]^−^	147.0429, 103.0532, 72.0079	l-phenylalanine	C_9_H_11_NO_2_	−4.86
6	8.431	131.0349	[M−H]^−^	87.0442	methylsuccinic acid	C_5_H_8_O_4_	0.63
6	9.156	315.0741	[M−H]^−^	153.0531, 108.0199	protocatechuic acid 4-glucoside (**P2**)	C_13_H_16_O_9_	−6.17
7	9.500	203.0837	[M−H]^−^	159.0903, 116.0483, 74.0237	(±)-tryptophan	C_11_H_12_N_2_O_2_	−5.41
8	10.127	285.0639	[M−H]^−^	153.0169, 108.0198	1-*O*-protocatechuyl-*β*-xylose (**P3**)	C_12_H_14_O_8_	−8.10
9	10.413	285.0635	[M−H]^−^	153.0171, 109.0276	1-*O*-protocatechuyl-*β*-xylose (**P3**)	C_12_H_14_O_8_	−6.70
9	10.476	325.0947	[M−H]^−^	163.0380, 119.0483	*trans*-*p*-coumaroyl-*β*-d-glucopyranoside (**P4**)	C_15_H_18_O_8_	−5.56
9	10.540	353.0904	[M−H]^−^	191.0540, 135.0426, 85.0277	caffeoylquinic acid (**QA1**)	C_16_H_18_O_9_	−7.35
10	10.595	158.0827	[M−H]^−^	116.0704	*N*-acetylvaline	C_7_H_13_NO_3_	−2.74
11	11.133	625.1444	[M−H]^−^	505.0937, 445.0717, 300.0251, 178.9957	quercetin 3-*O*-diglucoside (**Q1**)	C_27_H_30_O_17_	−5.40
11	11.154	315.1100	[M−H]^−^	162.0524	hydroxytyrosol 4′-*O*-glucoside (**P5**)	C_14_H_20_O_8_	−4.63
11	11.212	771.2019	[M−H]^−^	609.1352, 284.0298	kaempferol 3-*O*-*β*-d-diglucopyranoside 7-*O*-*β*-d-glucopyranoside (**K1**)	C_33_H_40_O_21_	−3.85
11	11.225	491.1436	[M + HCOO]^−^	413.1052, 293.0847, 149.0431, 89.0230	Monotropeoside (**P6**)	C_19_H_26_O_12_	−6.05
12	11.610	609.1500	[M−H]^−^	487.0977, 284.0295, 227.0334, 178.0038	kaempferol-7-sophoroside (**K2**)	C_27_H_30_O_16_	−6.39
12	11.677	337.0954	[M−H]^−^	191.0537, 93.0329	*p*-coumaroylquinic acid (**QA2**)	C_16_H_18_O_8_	−7.44
13	11.866	359.1007	[M + HCOO]^−^		2-hydroxyphenylacetic acid *O*-*β*-D-glucoside (**P7**)	C_14_H_18_O_8_	−6.49
13	12.009	367.1057	[M−H]^−^	191.0539, 93.0328	feruloylquinic acid (**QA3**)	C_17_H_20_O_9_	−6.11
14	12.126	933.2344	[M−H]^−^	787.1804, 665.1381, 300.0256	quercetin 3-(2-*p*-coumaroylsophoroside) 7-glucoside (**Q2**)	C_42_H_46_O_24_	−4.04
14	12.151	337.0948	[M−H]^−^	191.0538, 85.0508	*p*-coumaroylquinic acid (**QA2**)	C_16_H_18_O_8_	−7.44
14	12.189	771.1809	[M−H]^−^	609.1444, 383.0880, 285.0369	kaempferol 3-*O*-caffeoyl-sophoroside (**K3**)	C_36_H_36_O_19_	−4.02
14	12.446	801.1922	[M−H]^−^	625.1345, 300.0245, 225.1026	quercetin 3-*O*-feruloyl-sophoroside (**Q3**)	C_37_H_38_O_20_	−4.78
15	12.482	947.2510	[M−H]^−^	771.1867, 285.0338, 175.0355	kaempferol 3-*O*-feruloyl-sophoroside) 7-glucoside (**K4**)	C_43_H_48_O_24_	−4.99
15	12.510	771.1817	[M−H]^−^	625.1342, 300.0254, 178.9903	quercetin 3-*O*-coumaroyl-sophoroside (**Q4**)	C_36_H_36_O_19_	−5.05
15	12.758	917.2394	[M−H]^−^	771.1924, 631.1771, 284.0303	kaempferol 3-(2-*p*-coumaroylsophoroside) 7-glucoside (**K5**)	C_42_H_46_O_23_	−4.02
16	12.670	479.2532	[M + HCOO]^−^		unidentified		
16		547.2412			unidentified		
17	12.897	447.0959	[M−H]^−^	284.0299, 227.0318	kaempferol 7-*O*-glucoside (**K6**)	C_21_H_20_O_11_	−5.85
17	12.929	785.1976	[M−H]^−^	609.1348, 429.0687, 284.0311	kaempferol 3-*O*-feruloyl-sophoroside (**K7**)	C_37_H_38_O_19_	−5.28
17	12.992	755.1872	[M−H]^−^	681.3730, 609.1386, 367.0999, 284.0305	kaempferol 3-*O*-coumaroyl-sophoroside (**K8**)	C_36_H_36_O_18_	−5.71
17	13.302	755.1852	[M−H]^−^	609.1406, 284.0301	kaempferol 3-*O*-coumaroyl-sophoroside (**K8**)	C_36_H_36_O_18_	−3.06
17	13.528	163.0404	[M−H]^−^	119.0477	coumarinic acid	C_9_H_8_O_3_	−2.04
18	13.826	843.4786	[M + HCOO]^−^	797.4604, 635.4051	gymnemasaponin II (**T1**)	C_42_H_70_O_14_	−4.55
19	14.196	811.4555	[M−H]^−^	631.3809, 541.3826, 425.3342, 337.0772	(3*β*,16*β*)-16,28,29-trihydroxyolean-12-en-3-*yl*-2-*O*-*β*-d-glucopyranosyl-*β*-d-glucopyranosiduronic acid (**T2**)	C_42_H_68_O_15_	−8.57
19	14.396	827.4495	[M−H]^−^	783.4415, 647.3630, 603.3790, 113.0225, 59.0128	(3*β*,16*β*)-16,23,28,29-tetrahydroxyolean-12-*en*-3-*yl*-2-*O*-*β*-d-glucopyranosyl-*β*-d-glucopyranosiduronic acid (**T3**)	C_42_H_68_O_16_	−7.30
19	14.480	665.3933	[M+Cl]^−^		4-methoxycinnamoyloleanolic acid methyl ester (**T4**)	C_41_H_58_O_5_	6.80
19	14.746	811.4527	[M−H]^−^		(3*β*,16*β*)-16,28,29-trihydroxyolean-12-en-3-*yl*-2-*O*-*β*-d-glucopyranosyl-*β*-d-glucopyranosiduronic acid (**T2**)	C_42_H_68_O_15_	−5.12
20	14.860	957.5132	[M + HCOO]^−^	911.3846	28-[glucosyl-(1->6)-glucosyl] oleanolic acid 3-arabinoside (**T5**)	C_47_H_76_O_17_	−7.05
20		665.3942	[M+Cl]^−^	629.3686	4-methoxycinnamoyloleanolic acid methyl ester (**T4**)	C_41_H_58_O_5_	6.80
20		827.4483	[M−H]^−^		(3*β*,16*β*)-16,23,28,29-tetrahydroxyolean-12-*en*-3-*yl*-2-*O*-*β*-d-glucopyranosyl-*β*-d-glucopyranosiduronic acid (**T3**)	C_42_H_68_O_16_	−5.85
20	15.301	811.4553	[M−H]^−^	541.3830, 471.3374, 265.0995, 157.0113, 113.0229	(3*β*,16*β*)-16,28,29-trihydroxyolean-12-*en*-3-*yl*-2-*O*-*β*-d-glucopyranosyl-*β*-d-glucopyranosiduronic acid (**T2**)	C_42_H_68_O_15_	−8.32
21	15.473	665.3956	[M−H]^−^	555.9908, 489.3519, 423.3233, 307.0801	4-methoxycinnamoyloleanolic acid methyl ester (**T4**)	C_41_H_58_O_5_	3.35
21	15.516	795.458	[M−H]^−^		(3*β*,16*β*)-16,28-dihydroxyolean-12-*en*-3-yl-*O*-*β*-d-glucopyranosyl-*β*-d-glucopyranosiduronic acid (**T6**)	C_42_H_68_O_14_	−5.49
22	15.791	649.3994	[M−H]^−^		(3*β*,4α,16*β*)-16,23,28-trihydroxyolean-12-*en*-3-*yl*-2-*O*-*β*-d-glucopyranosiduronic acid (**T7**)	C_36_H_58_O_10_	−5.66
22	15.823	811.4528	[M−H]^−^	767.4590, 631.3771, 541.3836	(3*β*,16*β*)-16,28,29-trihydroxyolean-12-*en*-3-*yl*-2-*O*-*β*-d-glucopyranosyl-*β*-d-glucopyranosiduronic acid (**T2**)	C_42_H_68_O_15_	−5.24
23	16.289	797.4728	[M−H]^−^	635.4082	mabioside D (**T8**)	C_42_H_70_O_14_	−4.41
23	16.336	829.4978	[M + HCOO]^−^	783.4777, 621.4129	ginsenoside F2 (**T9**)	C_42_H_72_O_13_	−2.78
23	16.459	827.4831	[M + HCOO]^−^	781.4636, 619.4050, 545.5764, 161.0411	momordicoside D (**T10**)	C_42_H_70_O_13_	−3.93
23	16.537	795.4568	[M−H]^−^	615.3845, 525.3877, 157.0076, 89.0225	(3*β*,16*β*)-16,28-dihydroxyolean-12-*en*-3-yl-*O*-*β*-d-glucopyranosyl-*β*-d-glucopyranosiduronic acid (**T6**)	C_42_H_68_O_14_	−3.98
23	16.835	811.4542	[M−H]^−^	631.3778, 393.2268, 327.2136, 113.0211	(3*β*,16*β*)-16,28,29-trihydroxyolean-12-*en*-3-*yl*-2-*O*-*β*-d-glucopyranosyl-*β*-d-glucopyranosiduronic acid (**T2**)	C_42_H_68_O_15_	−6.97
24	16.945	795.4602	[M−H]^−^	615.3845, 525.3851, 157.0076, 89.0225	(3*β*,16*β*)-16,28-dihydroxyolean-12-*en*-3-yl-*O*-*β*-d-glucopyranosyl-*β*-d-glucopyranosiduronic acid (**T6**)	C_42_H_68_O_14_	−8.26
25	17.341	707.4050	[M−H]^−^	647.3531, 489.3479, 113.0222	hovenidulcioside B2 (**T11**)	C_38_H_60_O_12_	−5.37
26	17.611	707.4065	[M−H]^−^	647.3687, 489.3437	hovenidulcioside B2 (**T11**)	C_38_H_60_O_12_	−7.49
27	17.777	795.4610	[M−H]^−^	733.4316, 615.3834, 525.3877	(3*β*,16*β*)-16,28-dihydroxyolean-12-*en*-3-yl-*O*-*β*-d-glucopyranosyl-*β*-d-glucopyranosiduronic acid (**T6**)	C_42_H_68_O_14_	−9.26
28	18.530	649.3994	[M−H]^−^	617.3086, 441.3314, 293.2072, 175.0202, 113.0219	(3*β*,4α,16*β*)-16,23,28-trihydroxyolean-12-*en*-3-*yl*-2-*O*-*β*-d-glucopyranosiduronic acid (**T7**)	C_36_H_58_O_10_	−5.66
29	18.741	795.4587	[M−H]^−^	615.3799, 525.3842	(3*β*,16*β*)-16,29-dihydroxyolean-12-*en*-3-*yl*-2-*O*-*β*-d-glucopyranosyl-*β*-d-glucopyranosiduronic acid (**T12**)	C_42_H_68_O_14_	−9.26
30	19.427	681.4244	[M + HCOO]^−^	635.4093, 473.3468, 364.1692, 161.0439	triterpenoid (**T13**)	C_36_H_60_O_9_	−3.62
31	19.546	633.4049	[M−H]^−^	573.3726, 175.0236	(3*β*,16*β*)-16,29-dihydroxyolean-12-*en*-3-yl-*β*-d-glucopyranosiduronic acid (**T14**)	C_36_H_58_O_9_	−6.46
32	20.199	633.4059	[M−H]^−^	573.3738, 511.3683, 457.3599, 157.0117, 113.0225	(3*β*,16*β*)-16,29-dihydroxyolean-12-*en*-3-yl-*β*-d-glucopyranosiduronic acid (**T14**)	C_36_H_58_O_9_	−8.04
33	21.824	779.465	[M−H]^−^	717.4485, 599.3880, 509.3938, 157.0110	(3*β*,16*β*)-16-hydroxyolean-12-*en*-3-*yl*-2-*O*-*β*-d-glucopyranosyl-*β*-d-glucopyranosiduronic acid (**T15**)	C_42_H_68_O_13_	−8.06
34	21.968	721.3693	[M + HCOO]^−^	675.3528, 397.1311, 277.2145, 179.9526, 89.0228	gingerglycolipid A	C_33_H_56_O_14_	−5.67
35	22.343	798.4482	[M−H]^−^	647.3681, 175.0221, 113.0220	(3*β*,16*β*,22*α*)-22-(N-methylanthraniloxy)- 16,23,28-trihydroxyolean-12-*en*-3-yl-*O*-*β*-d-glucopyranosiduronic acid (**T16**)	C_44_H_65_NO_12_	−6.01
36	23.621	647.3316	[M + HCOO]^−^	601.3166, 341.1050, 323.0947, 277.2143, 179.0532, 89.0228	unidentified		
37	25.514	391.2628	[M−H]^−^	373.2473, 114.0177, 58.0287	3-hydroxy-10′-apo-*β*,*γ*-carotenal	C_27_H_36_O_2_	3.72
38	25.770	477.2651	[M−H]^−^	277.2162	schweinfurthin F	C_30_H_38_O_5_	−0.95
39	29.398	455.2799	[M−H]^−^	419.2983, 255.2310, 163.0584	unidentified	C_28_H_40_O_5_	0.87

The *m/z* of phenolic glycosides were found such as protocatechuic glycosides (compounds **P1 and P2**), *trans*-*p*-coumaroyl-*β*-d-glucopyranoside (compound **P3**), hydroxytyrosol 4′-*O*-glucoside (compound **P4**), monotropeoside (compound **P5**), 2-hydroxyphenylacetic acid *O*-*β*-D-glucoside (compound **P6**), and coumarinic acid (compound **P7**).

Possible molecules of quinic acids substituted with phenolic acid were detected. The compounds shared the MS/MS fragment of deprotonated quinic acid at *m/z* 191.054. Compound **QA1** gave ion products similar to caffeoylquinic acid (*m/z* 353.0904). The *m/z* of compound **QA2** was 30 a.m.u. lower than compound **QA3**. This suggested that compound **QA2** was *p*-coumaroylquinic acid whereas compound **QA3** was feruloylquinic acid.

The mass spectrometry also presented the ions of quercetin glycosides coupled with and without a phenolic component in the GIL samples. The compounds shared the structure of quercetin aglycone with the *m/z* of 300.025. Compound **Q1** could be quercetin 3-*O*-diglucoside ([M − H]^–^ with *m/z* 625.1444) based on the deduction of ion fragments [M−H−^0,2^X_1_]^−^ = 505.0937, [M−H–B_1_–H_2_O]^−^ = 445.0717, and [Y_0_–H]^•−^ = 300.0251 (see Fig. S3, Supplementary Material)Fig. S2 [32]. Compound **Q2** is referred to quercetin 3-(2-*p*-coumaroylsophoroside)-7-glucoside ([M − H]^–^ with *m/z* 933.2344) by giving the ion residue of [M−H−coumaryl]^−^ at *m/z* 787.1804. The detection of the [M−H−feruloyl]^−^ fragment ion (*m/z* 625.1345) on compound **Q3** ([M − H]^–^ with *m/z* 801.1922) can suggest the structure of quercetin 3-*O*-feruloyl-sophoroside. Meanwhile, the ion fragment of [M−H−coumaryl]^−^ at *m/z* 625.1342 also presented in compound **Q4,** which could be defined as quercetin 3-*O*-coumaroyl-sophoroside with *m/z* 771.1817.

Furthermore, the mass data of kaempferol glycosides were investigated. Kaempferol ions were observed in their MS/MS spectra at [M−2H]^•−^ with at *m/z* 284.030, or [M−H]^−^ with *m/z* at 285.040. Compound **K1** showed [M−H]^−^ at 771.2019 with a loss of 2−dehydroxy glucose (*m/z* 609.1352). It could be kaempferol 3-*O*-*β*-d-diglucopyranoside 7-*O*-*β*-d-glucopyranoside. The structure of compound **K2** (*m/z* 609.1500) can be identical to kaempferol-7-sophoroside, by referring [M−H–C_1_]^−^ ion equivalent to 429.0783 (see Fig. S4, Supplementary Material)Fig. S3 [32]. Compound **K3** (*m/z* 771.1809) can refer to kaempferol 3-*O*-caffeoyl-sophoroside by giving the ion of [M−H−caffeoyl]^−^ at 609.1444. Compound **K4** (*m/z* 947.2510) was tentatively identified as kaempferol 3-*O*-feruloyl-sophoroside 7-*O*-glucoside, by the evidence of the [M−H−feruloyl]^−^ ion at *m/z* 771.1867. Compound **K5** was proposed as kaempferol 3-(2-*p*-coumaroylsophoroside) 7-*O*-glucoside (*m/z* 917.2394) due to the fragment ions of *m/z* 771.1924 [M−H−coumaroyl]^−^ and 429.0793 [M−C_21_H_29_O_13_]^−^. In addition, compound **K6** could be a structure of kaempferol 7-*O*-glucoside by *m/z* at 447.0959. Compound **K7** could be kaempferol 3-*O*-feruloyl-sophoroside by the presence of a fragment ion with the loss of a feruloyl group, [M−H−feruloyl]^−^ equivalent to 609.1348. The mass of compound **K8** was less than compound **K7** by about 30 atomic mass unit (a.m.u.). Compound **K8** can be kaempferol 3-*O*-coumaroyl-sophoroside.

The mass ions of triterpenoids in the GIL samples were detected at the retention time between 13.826 min and 22.343 min. They might be gymnemasaponin II (compound **T1**, [M + HCOO]^−^ at 843.4786), (3*β*,16*β*)-16,28,29-trihydroxyolean-12-*en*-3-*yl*-2-*O*-*β*-d-glucopyranosyl-*β*-d-glucopyranosiduronic acid (compound **T2**, [M−H]^−^ at 811.4555) (Cpd6) [33], (3*β*,16*β*)-16,23,28,29-tetrahydroxyolean-12-*en*-3-*yl*-2-*O*-*β*-d-glucopyranosyl-*β*-d-glucopyranosiduronic acid (compound **T3**, [M−H]^−^ at 827.4495), 4-methoxycinnamoyloleanolic acid methyl ester (compound **T4**, [M−H]^−^ at 665.3933), 28-[glucosyl-(1->6)-glucosyl] oleanolic acid 3-arabinoside (compound **T5**, [M + HCOO]^−^ at 957.5132), (3*β*,16*β*)-16,28-dihydroxyolean-12-*en*-3-yl-*O*-*β*-d-glucopyranosyl-*β*-d-glucopyranosiduronic acid (compound **T6**, [M−H]^−^ at 795.458 and 795.4602) (GIA1) [15,25], (3*β*,4α,16*β*)-16,23,28-trihydroxyolean-12-*en*-3-*yl*-2-*O*-*β*-d-glucopyranosiduronic acid (compound **T7**, [M−H]^−^ at 649.3994) (GIA2) [25], mabioside D (compound **T8**, [M−H]^−^ at 797.4728), ginsenoside (compound **T9**, [M + HCOO]^−^ at 829.4978), momordicoside (compound **T10**, [M + HCOO]^−^ at 827.4831), hovenidulcioside B2 (compound **T11**, [M−H]^−^ at 707.4050 and 707.4065), (3*β*,16*β*)-16,29-dihydroxyolean-12-*en*-3-*yl*-2-*O*-*β*-d-glucopyranosyl-*β*-d-glucopyranosiduronic acid (compound **T12**, [M−H]^−^ at 795.4587), triterpenoid (compound **T13**, [M + HCOO]^−^ at 681.4244), (3*β*,16*β*)-16,29-dihydroxyolean-12-*en*-3-yl-*β*-d-glucopyranosiduronic acid (compound **T14**, [M−H]^−^ at 633.4049 and 633.4059), (3*β*,16*β*)-16-hydroxyolean-12-*en*-3-*yl*-2-*O*-*β*-d-glucopyranosyl-*β*-d-glucopyranosiduronic acid (compound **T15**, [M−H]^−^ at 779.4650) (Cpd5) [33], (3*β*,16*β*,22*α*)-22-(N-methylanthraniloxy)- 16,23,28-trihydroxyolean-12-*en*-3-yl-*O*-*β*-d-glucopyranosiduronic acid (compound **T16**, [M−H]^−^ at 798.4482) (Cpd 8 and GIA7) [21,33].

Other metabolites could be amino acids; *N*-(1-deoxy-1-fructosyl) alanine, *N*-acetylvaline, phenylalanine, and tryptophan, sugars; mannitol, sucrose, and hexose, other metabolites (e.g., malic acid, methylsuccinic acid, ginger glycolipid A, 3-hydroxy-10′-apo-*β*,*γ*-carotenal, and schweinfurthin F), and unidentified compounds.

### 3.2 Phytochemical quantification by authentic standards

The *G. inodorum* leaf samples (GIL1 to GIL5) from five collection areas contained different amounts of total phenolics (52.58–126.19 mg GAE/g extract), total flavonoids (59.62–133.43 mg QE/g extract) and triterpene saponins (89.23–204.23 mg UAE/g extract) (see Table 2).

**Table 2:** Quantification of phenolics, flavonoids, and triterpene saponins in *G. inodorum* leaf extracts.

Samples	TPCs (mg GAE/g extract)	TFCs (mg QE/g extract)	TTCs (mg UAE/g extract)	Contents (mg/g extract)			
				Kaempferol	Quercetin	Isorhamnetin	GIA1
**GIL1**	52.58 ± 3.64^d^	66.76 ± 7.33^b^	163.02 ± 0.09^b^	0.628 ± 0.008^d^	0.247 ± 0.002^d^	0.005 ± 0.000	169.6 ± 11.4^a^
**GIL2**	66.27 ± 6.90^c^	59.62 ± 3.30^b^	204.23 ± 4.82^a^	12.624 ± 1.552^c^	6.428 ± 0.272^c^	0.009 ± 0.000	182.7 ± 11.0^a^
**GIL3**	98.81 ± 5.89^b^	85.33 ± 2.97^b^	89.23 ± 4.82^d^	33.532 ± 2.218^b^	17.172 ± 0.498^b^	0.004 ± 0.000	53.4 ± 2.4^b^
**GIL4**	112.50 ± 6.55^b,a^	133.43 ± 3.78^a^	126.50 ± 6.11^c^	76.284 ± 2.278^a^	24.535 ± 0.076^a^	0.007 ± 0.000	72.5 ± 11.4^b,c^
**GIL5**	126.19 ± 4.49^a^	131.52 ± 2.97^a^	113.32 ± 1.61^c^	81.483 ± 4.433^a^	25.401 ± 0.011^a^	0.007 ± 0.001	63.9 ± 1.4^c^

a, b, c, and d represent the significant difference between groups at *p* value ≤ 0.05. TPCs, total phenolic contents; TFCs, total flavonoid contents; TTCs, total triterpenoid contents; GIA1, (3*β*, 16*β*)-16,28-dihydroxyolean-12-*en*-3-yl-*O*-*β*-*d*-glucopyranosyl-*β*-*d*-glucopyranosiduronic acid.

The phenolic compounds in *G. inodorum* leaf samples were authenticated and quantified compared to the chemical standards of phenolics and flavonoids (see Materials and Methods). This study found the flavonoid glycosides were derived from quercetin and kaempferol. The samples (GIL1 to GIL5) had quercetin at 0.247 ± 0.002, 6.428 ± 0.272, 17.172 ± 0.498, 24.535 ± 0.076 and 25.401 ± 0.011 mg/g extract, and kaempferol at 0.628 ± 0.008, 12.624 ± 1.552, 33.532 ± 2.218, 76.284 ± 2.278 and 81.483 ± 4.433 mg/g extract, respectively. The small amounts of isorhamnetin were observed to be about 0.004–0.007 mg/g extract.

Compound **T6** had the ion fragmentations identical to (3*β*,16*β*)-16,28-dihydroxyolean-12-*en*-3-yl-*O*-*β*-d-glucopyranosyl-*β*-d-glucopyranosiduronic acid (GIA1). It was purified from the GIL1 sample from our previous study [15]. The GIA1 compositions were found in the GIL1 to GIL5 samples at 169.6 ± 11.4, 182.7 ± 11.0, 53.4 ± 2.4, 72.5 ± 11.4 and 63.9 ± 1.4 mg/g extract, respectively.

Overall, the GIL1, GIL2 and GIL3 samples tended to contain lower phenolics and higher triterpenoid content than GIL4 and GIL5. These results are shown in Table 2.

### 3.3 Antioxidations of *G. inodorum* leaves

Antioxidant capacities of the *G. inodorum* leaf samples were measured and compared to Trolox equivalent (TE) capacities. The results are shown in Table 3. The antioxidant capacities of GIL1 to GIL5 were 54.43 ± 0.14, 66.17 ± 0.06, 113.49 ± 0.89, 137.46 ± 0.83, and 135.06 ± 0.80 μmol TE/g extract for scavenging DPPH radicals, and 67.89 ± 0.10, 136.15 ± 0.58, 159.99 ± 0.55, 215.79 ± 1.10, and 249.75 ± 0.90 μmol TE/g extract for neutralizing ABTS radicals. The FRAP method indicated the antioxidant capacities of GIL1 to GIL5 at 59.06 ± 0.34, 72.05 ± 1.89, 96.14 ± 1.69, 117.83 ± 7.77, and 119.36 ± 2.99 μmol TE/g extract. The ORAC assay showed the antioxidant capacities at 455.96 ± 12.65, 558.27 ± 49.99, 840.61 ± 27.99, 1843.45 ± 75.22, and 2239.73 ± 51.89 μmol TE/g extract.

**Table 3:** Antioxidant capacities of *G. inodorum* leaf extracts by DPPH, ABTS, FRAP, and ORAC assays.

Samples	Antioxidant capacities (μmol TE/g extract)			
	DPPH	ABTS	FRAP	ORAC
**GIL1**	54.43 ± 0.14^d^	67.89 ± 0.10^e^	59.06 ± 0.34^d^	455.96 ± 12.65^c^
**GIL2**	66.17 ± 0.06^c^	136.15 ± 0.58^d^	72.05 ± 1.89^c^	558.27 ± 49.99^c^
**GIL3**	113.49 ± 0.89^b^	159.99 ± 0.55^c^	96.14 ± 1.69^b^	840.61 ± 27.99^b^
**GIL4**	137.46 ± 0.83^a^	215.79 ± 1.10^b^	117.83 ± 7.77^b,a^	1843.45 ± 75.22^a^
**GIL5**	135.06 ± 0.80^a^	249.75 ± 0.90^a^	119.36 ± 2.99^a^	2239.73 ± 51.89^a^

a, b, c, d, and e represent the significant difference between groups at *p* value ≤ 0.05. DPPH; 2,2-diphenyl-1-picrylhydrazyl radical scavenging assay, ABTS; 2,2′-azinobis (3-ethylbenzo-thiazoline-6-sulfonic acid) radical cation-based scavenging assay, FRAP; ferric reducing antioxidant power assay, ORAC; oxygen radical antioxidant capacity assay. μmol TE/g extract is the unit of micromolar Trolox equivalent per 1 g extract.

The statistical results indicated the characteristics of phytochemicals and antioxidant activities between the *G. inodorum* leaf samples collected from five different areas. The samples of GIL1, GIL2 and GIL3 seemed to significantly differ between groups in terms of phytochemical distributions and antioxidations. Meanwhile, the GIL4 and GIL5 samples showed comparable results for their phytochemicals and antioxidant characteristics.

### 3.4 Relations of phytonutrients and antioxidation capacities of *G. inodorum* leaves

The relationship of the G. *inodorum* leaf phytochemicals and their antioxidation capacities was studied by Pearson's correlation coefficients. The results are presented in Table 4. Positive values of the correlation coefficients (r) closed to 1 were observed in between the variables, such as 1) the *G. inodorum* leaf phytonutrients (including total phenolics, total flavonoids, kaempferol, quercetin and isorhamnetin) vs. the antioxidant activities by DPPH, ABTS, FRAP and ORAC methods, 2) total phenolics and total flavonoids vs. kaempferol and quercetin, 3) total triterpenoids vs. GIA1, and 4) among the antioxidant activities. The r coefficients were found at negative values between −0.5 and −1 within the variable groups, such as 1) total phenolics and total flavonoids vs. total triterpenoids and GIA1, and 2) total triterpenoids and GIA1 vs. the antioxidant activities.

**Fig. 1:**
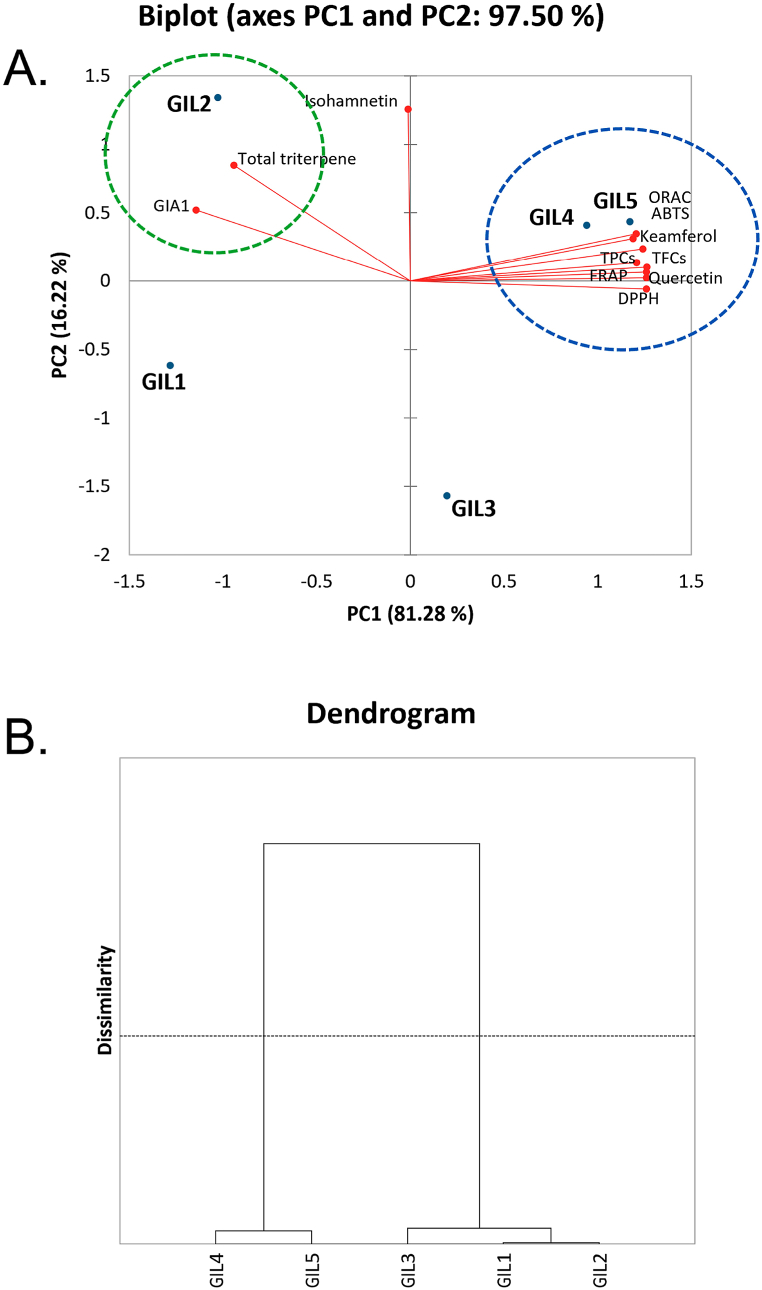
(A) Principal component analysis (PCA) biplot, and (B) hierarchical cluster analysis (HCA) of *G. inodorum* extracts (GIL1 to GIL5) based on ten studies; TPCs (total phenolic contents), TFCs (total flavonoid contents), kaempferol, quercetin, isorhamnetin and GIA1 contents, and antioxidant activities using DPPH, ABTS, FRAP, and ORAC methods.

We employed the advantages of principal component analysis (PCA) to summarize the numerical data into an interpretable figure. Thus, the mean values of TPCs, TFCs, TTCs, quercetin, kaempferol, isorhamnetin, GIA1, and antioxidant activities (DPPH, ABTS, FRAP and ORAC) were used in the PCA analysis. Fig. 1A shows the biplot consisting of PC1 and PC2. The combination of PC1 (81.28 %) and PC2 (16.22 %) was 97.50 %. This indicates that the biplot represents 97.50 % of the input data, indicating that it is an adequate representation for interpretation. The PC1 axis contains most of the data, except for TTCs isorhamnetin and GIA1, which were placed on the PC2 axis. Fig. 1A clearly shows that GIL4 and GIL5 were clustered with analyzed variables kaempferol, quercetin, TFCs, TPCs and antioxidant activities (DPPH, ABTS, FRAP, and ORAC) while GIL2 was clustered with GIA1 and TTCs and GIL1 and GIL3 distributed randomly in the biplot. This indicates that GIL4 and GIL5 had strong relationships with clustered variables (blue circle in Fig. 1), while GIL2 had relationship with GIA1 and TTCs and GIL1, and GIL3 had poor relationship with all variables. Interestingly, all samples were located far away from isorhamnetin, confirming the data in Table 4 that all samples had a poor relationship with this compound. This could be due to the extremely low amount of isorhamnetin in GIL samples. In summary, the PCA implies that both GIL4 and GIL5 had high correlations with kaempferol, quercetin, TFCs, TPCs, and antioxidant activities and GIL2 had a high correlation with GIA1 and total triterpene.

**Table 4:** Pearson's correlation coefficients (r) between total phenolic contents (TPCs), total flavonoid contents (TFCs), total triterpenoid contents (TTCs), GIA1, kaempferol, quercetin, and isorhamnetin in *G. inodorum* extracts and their antioxidation activities from DPPH, ABTS, FRAP, and ORAC assays.

	TPCs	TFCs	TTCs	GIA	Kaempferol	Quercetin	Isorhamnetin	DPPH	ABTS	FRAP	ORAC
**TPCs**	1										
**TFCs**	0.915	1									
**TTCs**	−0.733	−0.619	1								
**GIA**	−0.899	−0.778	0.947	1.000							
**Kaempferol**	0.963	0.979	−0.595	−0.791	1						
**Quercetin**	0.991	0.924	−0.702	−0.888	0.969	1					
**Isorhamnetin**	0.020	0.057	0.662	0.393	0.161	0.059	1				
**DPPH**	0.982	0.928	−0.764	−0.924	0.956	0.993	−0.043	1			
**ABTS**	0.965	0.889	−0.531	−0.759	0.963	0.964	0.280	0.928	1		
**FRAP**	0.988	0.944	−0.684	−0.872	0.982	0.998	0.077	0.991	0.966	1	
**ORAC**	0.923	0.971	−0.528	−0.712	0.981	0.912	0.199	0.892	0.937	0.932	1

A dark to light gray color represents the Pearson's correlation coefficients (r) ranging from 1 to −1.

The sample clustering, which may anticipate the association between each sample, can be computed based on the sample mean data. Hierarchical cluster analysis (HCA) was used for this objective. The results revealed that five GIL samples were separated into two groups. The first group contained GIL4 and GIL5 which exhibited high phenolic contents and antioxidant activities, while GIL1, GIL2 and GIL3 were clustered together because they were low in phenolic compounds and antioxidant effects (Fig. 1). Thus, both PCA and HCA confirm the close cluster of GIL4 and GIL5, suggesting the linear correlation between phenolic contents and antioxidations of the GIL plant.

### 3.5 Cellular antioxidant activity of G. inodorum leaves on reactive nitrogen species using HUVEC model

**Fig. 2:**
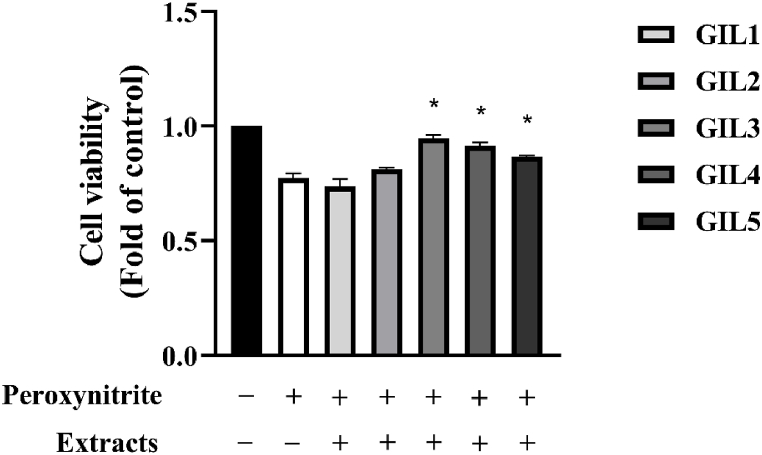
Cell viability of human umbilical vein endothelial cells (HUVEC) induced an oxidative stress by 14.5 μM peroxynitrite with and without the pre-treatment of 1.95 μg/mL G*. inodorum* extracts (GIL1 to GIL5). The * symbol represents significant cell recovery at *p* values ≤ 0.05 as compared to a peroxynitrite treated group.

Peroxynitrite (ONOO^−^) at 14.5 μM induced oxidative stress on human umbilical vein endothelial cells (HUVEC). It caused about 20 % of the cell death. Interestingly, the HUVEC vitality tended to be recovered when the cells were pre-treated with the non-toxic dose of GIL1 to GIL5 at 1.95 μg/mL before being exposed to ONOO^−^. The GIL3, GIL4 and GIL5 samples significantly maintained the HUVEC viability of about 90 %–95 % in the oxidative condition. Meanwhile, GIL1 and GIL2 had fewer antioxidant effects. The results are presented in Fig. 2.

## 4 Discussion

*G. inodorum* was reported as an antioxidant food [22,26], but its effective phytonutrients have not been investigated. This recent study observed possible *m/z* ions that may involve the antioxidant functions of the *G. inodorum* leaf (GIL) samples by tandem mass spectrometry. The mass analysis suggested the phytochemical structures of phenolic acid, phenolic glycosides, quinic acids, quercetin glycosides, kaempferol glycosides, and triterpenoid glycosides. The reference standards of phenolics, quinic acids, flavonoids, and GIA1 were used to authenticate the GIL phytochemicals. Quercetin and kaempferol were flavonoids in the GIL samples containing about 0.247–25.401 mg quercetin/g extract and about 0.628–81.483 mg kaempferol/g extract. A small amount of isorhamnetin was detected, but it was less than 0.001 mg/g extract. This study also confirmed GIA1 as one of the triterpenoid glycosides. The GIL samples had about 53.4–182.7 mg GIA1/g extract. The other triterpenoid molecules were predicted by the mass database, but they were not observed in this study due to the unavailability of the chemical standards. However, the differences in the amounts of quercetin, kaempferol and GIA1 among the GIL samples are possible from agricultural factors such as cultivation area, climate, harvesting protocol and harvesting time [34,35].

Furthermore, the GIL phytochemicals showed the antioxidant activities related to two mechanisms: hydrogen atom transfer (HAT) and electron transfer (ET). The antioxidant assays were performed by DPPH, ABTS, FRAP, and ORAC methods [36,37]. The antioxidant activities of the GIL samples in this recent study were comparable to works of Dunkhunthod et al. [18] and Jeytawan et al. [19]. In this study, the Pearson's correlation coefficients (*r*) indicated the strong relations of quercetin and kaempferol contents as the major flavonoid components in the GIL phytochemicals to exhibit the antioxidant activities. Meanwhile isorhamnetin, TTCs, and GIA1 in the GIL samples showed a low antioxidant effect. The PCA and HCA results also confirmed the GIL samples' classification into two groups. The first group contained high phenolic contents and antioxidant activities, represented by the GIL4 and GIL5 samples, while the second group (GIL1, GIL2 and GIL3) with low in phenolic contents and antioxidant activities were clustered together. Thus, it can be inferred from the results of Pearson's correlation coefficients, PCA and HCA that the GIL materials with a high-quality antioxidant potential should have phenolic and flavonoid contents higher than triterpenoid compositions.

The cellular antioxidant activity of the GIL phytonutrients was also carried out on human umbilical vein endothelial cells (HUVEC) which induced dysfunction and injury by peroxynitrite (ONOO^−^). It is a highly reactive oxidant found in the early stage of diabetic complications generated at high levels by glucose oxidation. Endothelial cells exposed to peroxynitrite at a long period of time will lead to cell abnormality which can readily further develop vascular diseases and diabetic complications [38,39]. Our experimental results showed that that the GIL samples containing high phenolic contents (GIL3, GIL4 and GIL5) significantly recovered the viability of HUVEC upon oxidative stress. These are promising results to develop *G. inodorum* as an antioxidant functional ingredient for anti-diabetic complications support. The biochemical mechanisms on the cellular antioxidant activity of the GIL phytonutrients will be further explained in terms of gene expression related to the cell viability of HUVEC cultured with and without peroxynitrite. In our future work, the cellular antioxidant functions of the GIL phytonutrients will also be compared to their reference standards, such as kaempferol, quercetin and GIA1.

Moreover, the availability of triterpenoid compositions could give other anti-diabetic supports, such as anti-inflammation [[18], [19], [20]], anti-insulin resistant and mimetic activities [20,33], inhibiting α-amylase and α-glucosidase actions [15,16], reducing intestinal glucose absorption [15,17], and anti-adipocyte differentiation [21]. Thus, the consumption of *G. inodorum* leafy green vegetables can offer variations of the GIL phytonutrients for diabetic prevention together with anti-diabetic complications. The health-promoting properties of *G. inodorum* may also depend on the raw material's phytochemical compositions.

## 5 Conclusion

Tandem mass spectrometry indicated the mass signals of tentative phytonutrients in the *G. inodorum* leaves, such as phenolics, quinic acids, flavonoids, and triterpenoid saponins. Using reference standards, quercetin, kaempferol and GIA1 were authenticated as flavonoid and triterpene in this plant. This study did not detect other phenolic acids, quinic acids, or triterpenes. *G. inodorum* leaf extracts showed antioxidant properties via hydrogen atom and electron transfers. The antioxidant effects had strong relations to phenolics, flavonoids, quercetin and kaempferol, but low correlations to triterpenoids and GIA1. The leaf samples with high contents of phenolics, flavonoids, quercetin, and kaempferol showed a significant outcome on the cellular antioxidation by the observation on HUVEC which induced cell death by peroxynitrite. The viability of HUVEC in the oxidative stress were recovered similar to normal cells. The *G. inodorum* vegetable is a promising plant material to develop as an antioxidant functional food to prevent diabetic complications and other non-communicable diseases.

## Data availability

Data included in article/supplement material/referenced in article.

## Ethics statement

Not applicable.

## CRediT authorship contribution statement

**Onanong Nuchuchua:** Writing - review & editing, Writing - original draft, Visualization, Validation, Supervision, Software, Resources, Project administration, Methodology, Investigation, Funding acquisition, Formal analysis, Data curation, Conceptualization. **Wanwisa Srinuanchai:** Writing - review & editing, Writing - original draft, Visualization, Validation, Software, Resources, Methodology, Investigation, Formal analysis, Data curation. **Chaisak Chansriniyom:** Writing - review & editing, Writing - original draft, Validation, Methodology, Investigation, Formal analysis, Data curation, Conceptualization. **Uthaiwan Suttisansanee:** Writing - review & editing, Writing - original draft, Visualization, Validation, Supervision, Methodology, Investigation, Formal analysis. **Piya Temviriyanukul:** Writing - review & editing, Writing - original draft, Validation, Software, Methodology, Formal analysis, Data curation, Conceptualization. **Nitra Nuengchamnong:** Writing - review & editing, Validation, Software, Methodology, Investigation, Formal analysis, Data curation. **Uracha Ruktanonchai:** Writing - review & editing, Visualization, Supervision.

## Declaration of competing interest

The authors declare that they have no known competing financial interests or personal relationships that could have appeared to influence the work reported in this paper.
