# Supplementary material for: Relationship of phytochemicals and antioxidant activities in Gymnema inodorum leaf extracts
Source: Heliyon. 2023 Dec 2;10(1):e23175. doi: 10.1016/j.heliyon.2023.e23175 (PMC10755283; doi:10.1016/j.heliyon.2023.e23175)
Supplement: Multimedia component 1 [file mmc1.docx]

**Supplementary material**

**Relationship of phytochemicals and antioxidant activities in *Gymnema inodorum* leaf extracts**

Onanong Nuchuchua^a,*^, Wanwisa Srinuanchai^a^, Chaisak Chansriniyom^b,c^, Uthaiwan Suttisansanee^d^, Piya Temviriyanukul^d^, Nitra Nuengchamnong^e^, Uracha Ruktanonchai^f^

^a^National Nanotechnology Center (NANOTEC), National Science and Technology Development Agency (NSTDA), Pathum Thani, Thailand

^b^Department of Pharmacognosy and Pharmaceutical Botany, Faculty of Pharmaceutical Sciences, Chulalongkorn University, Bangkok, Thailand

^c^Natural products and Nanoparticles Research Unit, Chulalongkorn University, Bangkok, Thailand

^d^Institute of Nutrition, Mahidol University, Nakhon Pathom, Thailand

^e^Science Laboratory Center, Faculty of Science, Naresuan University, Phitsanulok, Thailand

^f^National Science and Technology Development Agency (NSTDA), Pathum Thani, Thailand

* Corresponding author: Nano Agricultural Chemistry and Processing Research Team, National Nanotechnology Center, National Science and Technology Development Agency.

Tel.: +66 2 117 6792

E-mail address: [onanong@nanotec.or.th](mailto:onanong@nanotec.or.th)

**Table S1.** *G. inodorum* leaf specimens in this study collected from five different areas and their phenolic contents.

| **Sample details** | **GIL1*** | **GIL2** | **GIL3** | **GIL4** | **GIL5** |
| --- | --- | --- | --- | --- | --- |
| Collection Areas | Chaopraya Abhaiphubejhr Hospital, Prachin Buri Province, Thailand | Chiang-Da farming group, Fang District, Chiang Mai Province, Thailand | Home-grown garden, Chiang-Rai Province, Thailand | Chiang-Da Gathong, San Mahaphon, Mae Taeng District, Chiang Mai Province, Thailand | Fresh market, Chiang-Rai Province, Thailand |
| Collection dates | August 2018 | August 2020 | August 2020 | October 2018 | September 2020 |
| Voucher herbarium  specimens | BK no. 171395, deposited at the Plant Varieties Protection Office, Department of Agriculture, Ministry of Agriculture and Cooperatives, Thailand | No. 38430, deposited at the herbarium of Chiang Mai University (CMU) herbarium and flora database, Department of Biology, Faculty of Science, Chiang Mai University, Thailand | BK No. 070339, deposited at the Plant Varieties Protection Office, Department of Agriculture, Ministry of Agriculture and Cooperatives, Thailand | − | − |

*Plant specimen published in Srinuanchai et al., 2021.


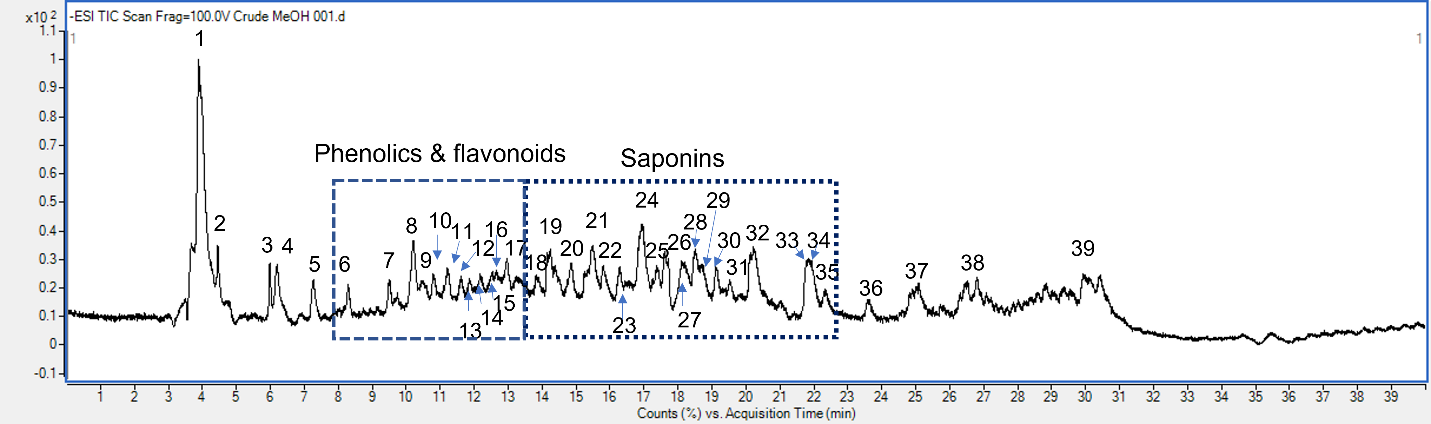


**Fig. S1.** Total ion chromatogram of *G. inodorum* extract using a negative ionization mode. The peak numbers are referred to the compounds listed in Tables 3.


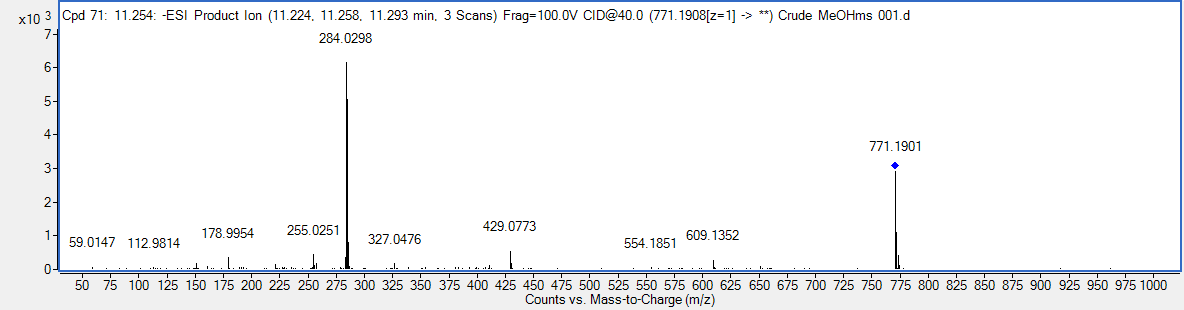

**Compound K1**

**
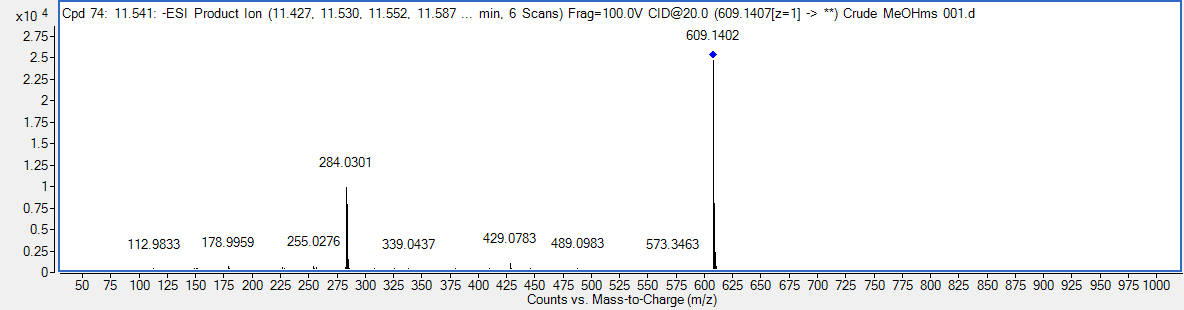
**

**
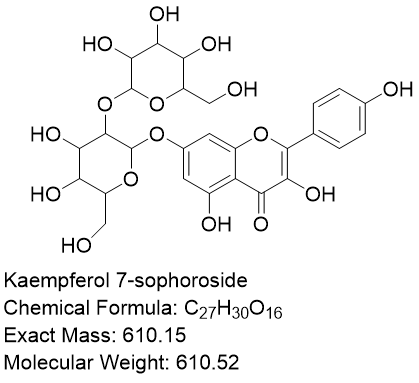
**

**Compound K2**


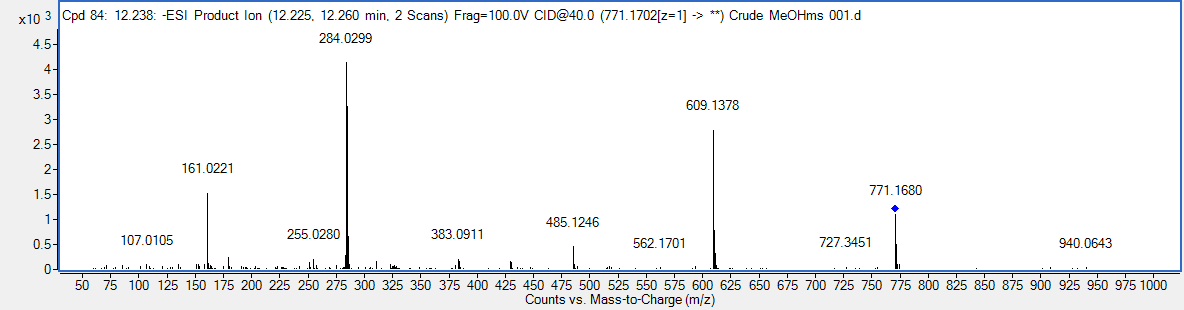

**Compound K3**

**
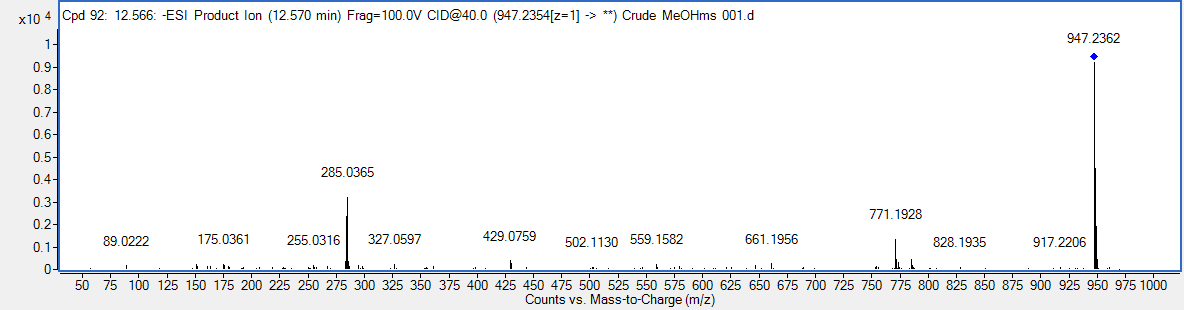
**

**Compound K4**


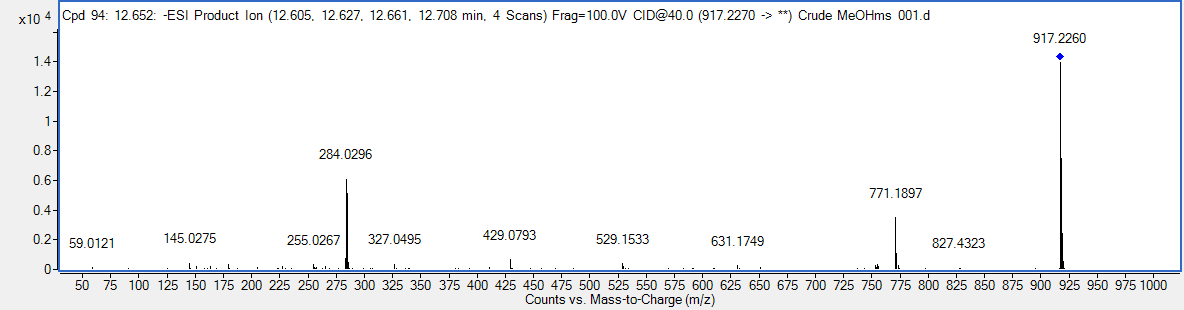

**Compound K5**

**
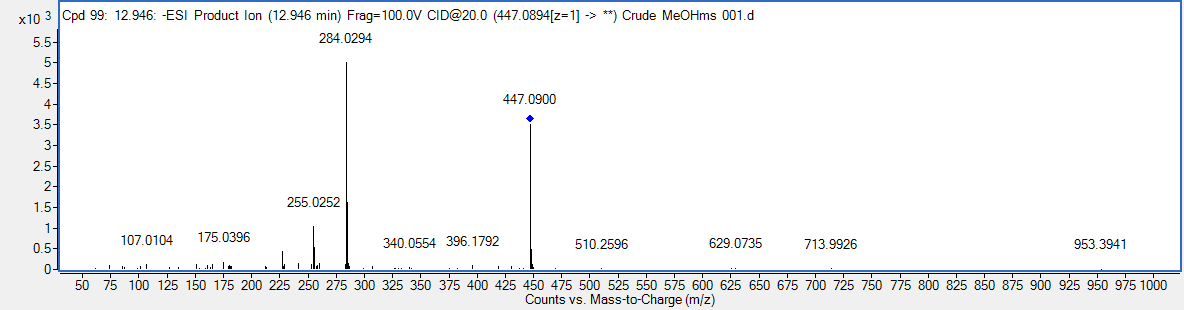
**

**
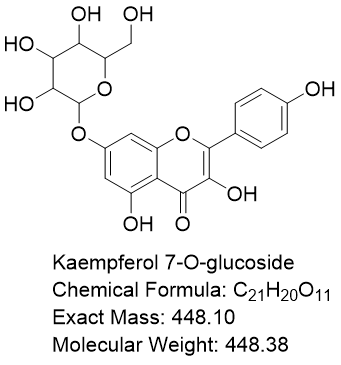
**

**Compound K6**
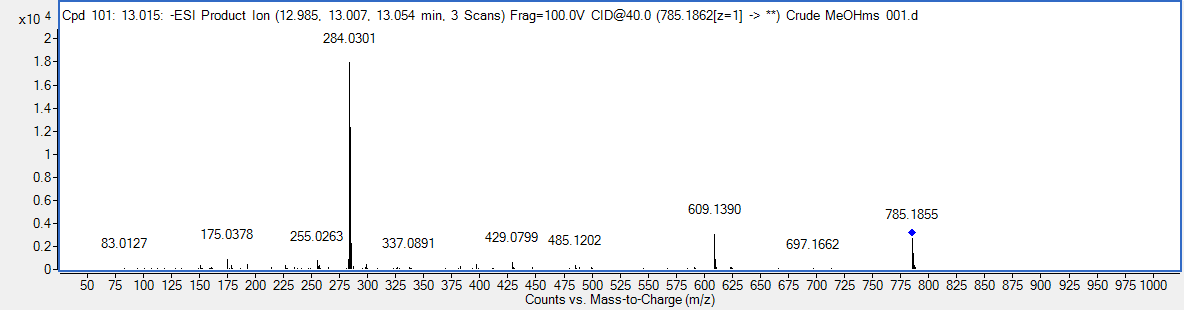

**Compound K7**


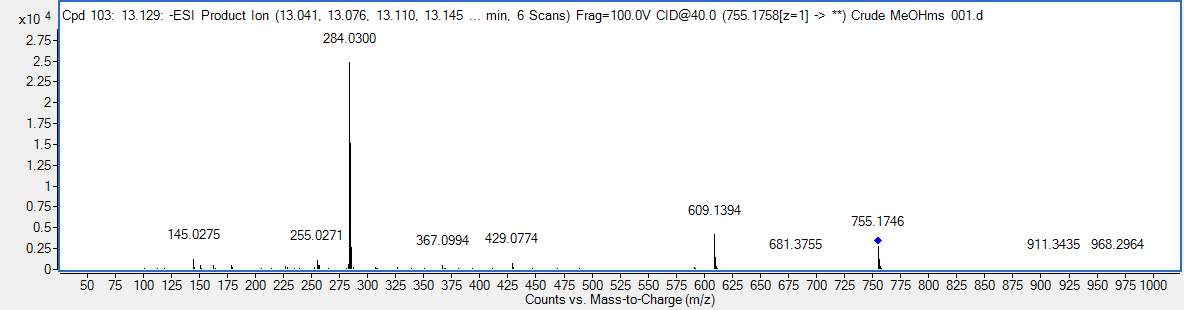

**Compound K8**

**
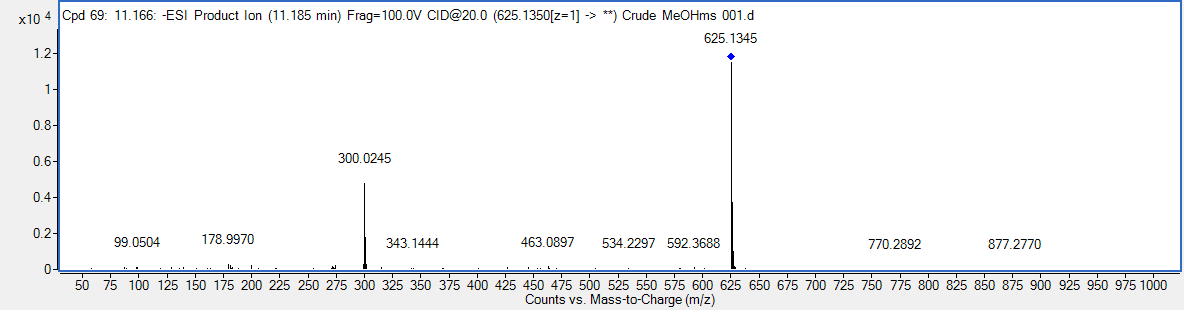
**

**Compound Q1**

**
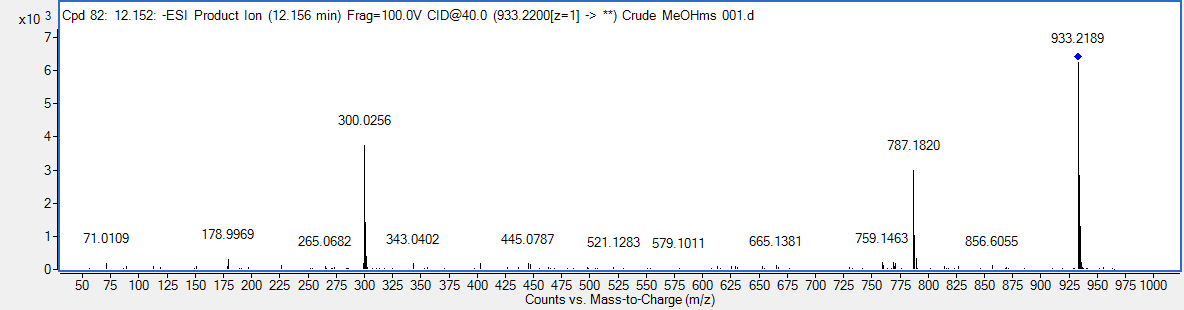
**

**Compound Q2**

**
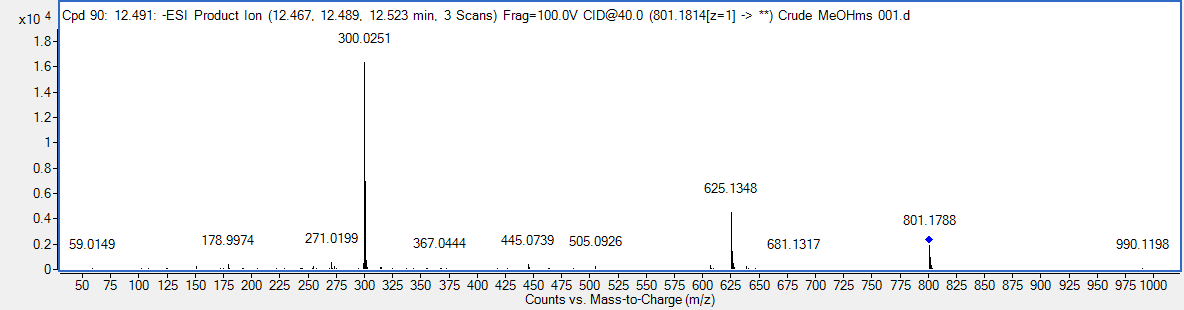
**

**Compound Q3**

**
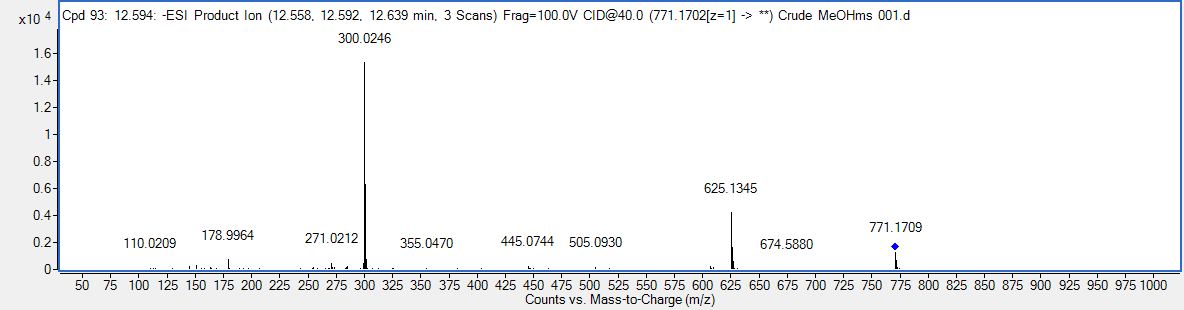
**

**Compound Q4**


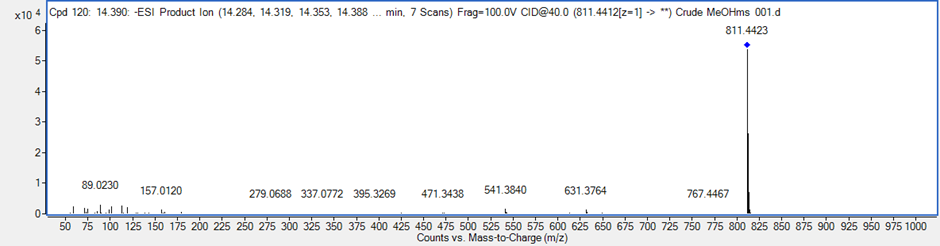


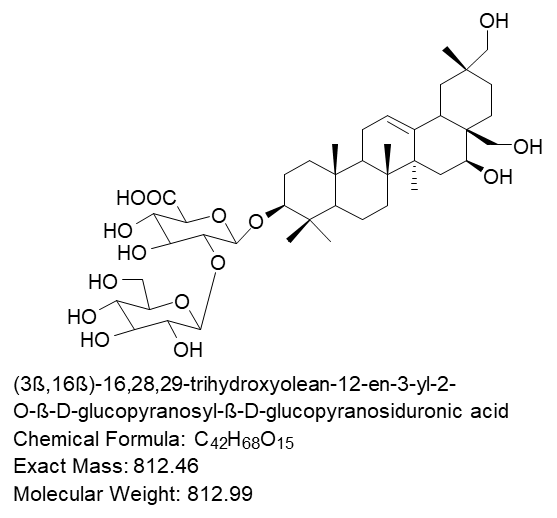


**Compound T2**

**
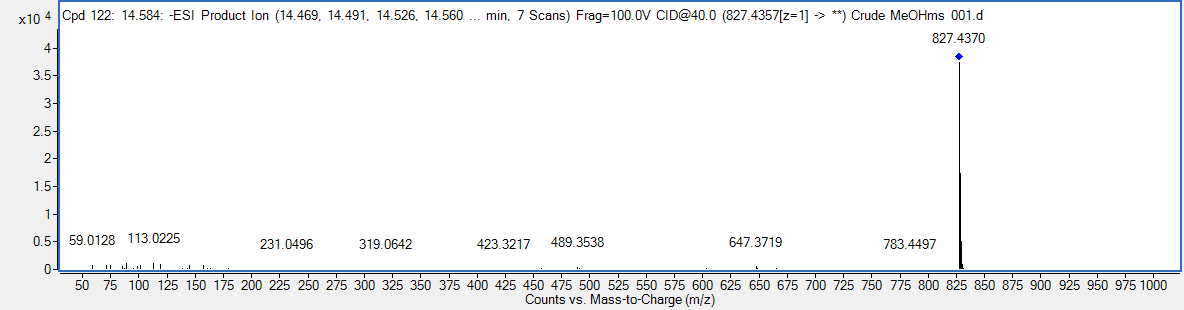
**


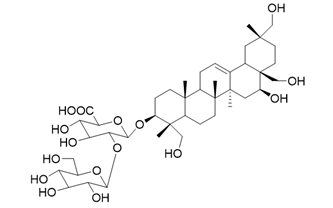


**Compound T3**

**
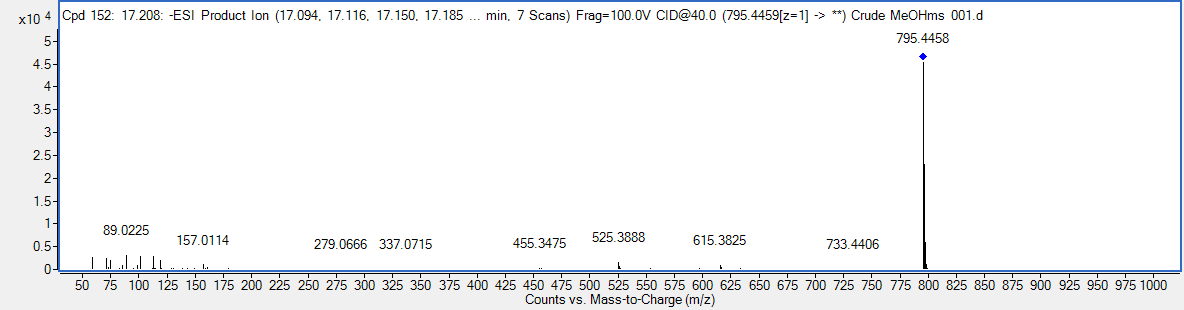
**

**Compound T6**

**
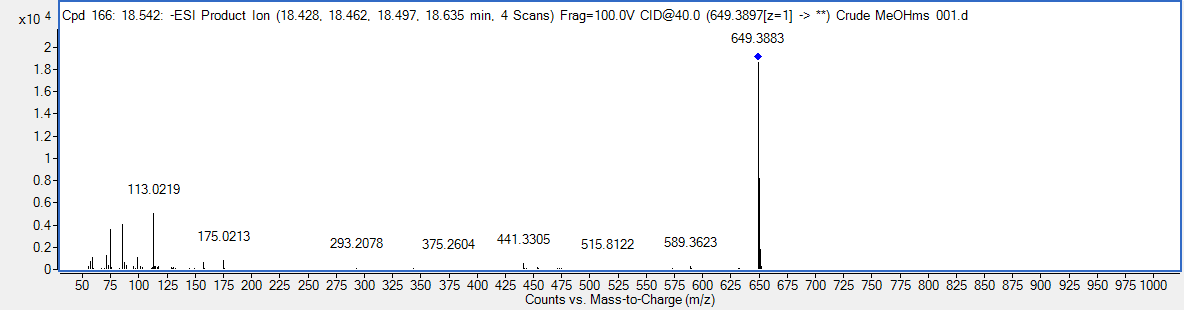
**

**Compound T7**

**
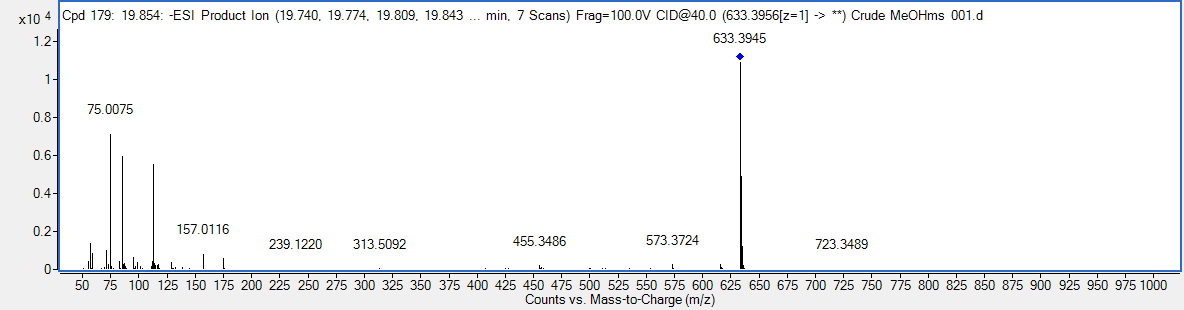
**


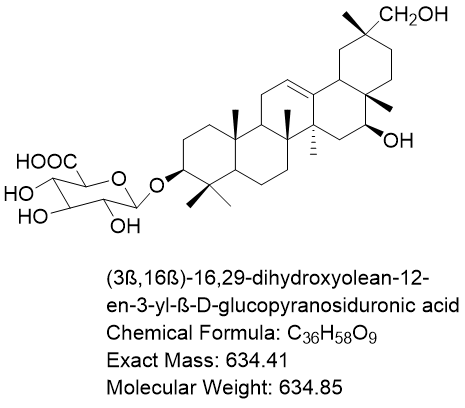


**Compound T14**

**
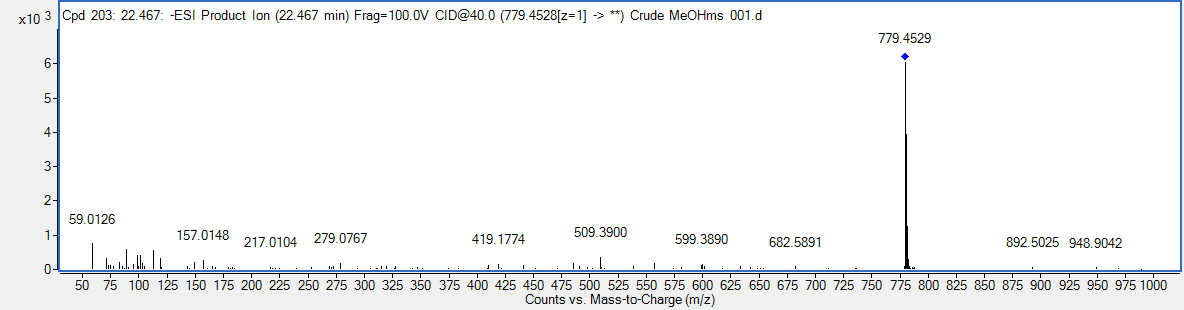
**

**
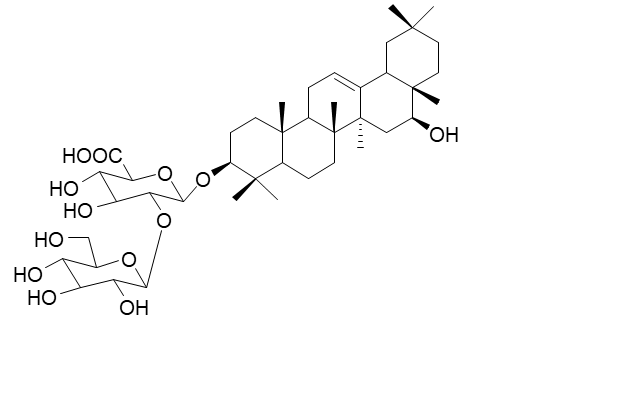
**

**Compound T15**

**Fig. S2.** Ion fragmentations and chemical structures of tentative *G. inodorum* phytonutrients, such as (a) kaempferol 3-*O*-*β*-D-diglucopyranoside 7-*O*-*β*-D-glucopyranoside (**K1**), (b) kaempferol-7-sophoroside (**K2**), (c) kaempferol 3-*O*-caffeoyl-sophoroside (**K3**), (d) kaempferol 3-*O*-feruloyl-sophoroside) 7-glucoside (**K4**), (e) kaempferol 3-(2-*p*-coumaroylsophoroside) 7-glucoside (**K5**), (f) kaempferol 7-*O*-glucoside (**K6**), (g) kaempferol 3-*O*-feruloyl-sophoroside (**K7**), (h) kaempferol 3-*O*-coumaroyl-sophoroside (**K8**), (i) quercetin 3-*O*-diglucoside (**Q1**) , (j) quercetin 3-(2-*p*-coumaroylsophoroside) 7-glucoside (**Q2**), (k) quercetin 3-*O*-feruloyl-sophoroside (**Q3**), (l) quercetin 3-*O*-coumaroyl-sophoroside (**Q4**), (m) (3*β*,16*β*)-16,28,29-trihydroxyolean-12-en-3-*yl*-2-*O*-*β*-D-glucopyranosyl-*β*-D-glucopyranosiduronic acid (**T2**), (n) (3*β*,16*β*)-16,23,28,29-tetrahydroxyolean-12-*en*-3-*yl*-2-*O*-*β*-D-glucopyranosyl-*β*-D-glucopyranosiduronic acid (**T3**), (o) (3*β*,16*β*)-16,28-dihydroxyolean-12-*en*-3-*yl*-*O*-*β*-D-glucopyranosyl-*β*-D-glucopyranosiduronic acid (**T6**), (p) (3*β*,4α,16*β*)-16,23,28-trihydroxyolean-12-*en*-3-*yl*-2-*O*-*β*-D-glucopyranosiduronic acid (**T7**), (q) (3*β*,16*β*)-16,29-dihydroxyolean-12-*en*-3-*yl*-*β*-D-glucopyranosiduronic acid (**T14**), (r) (3*β*,16*β*)-16-hydroxyolean-12-*en*-3-*yl*-2-*O*-*β*-D-glucopyranosyl-*β*-D-glucopyranosiduronic acid (**T15**).


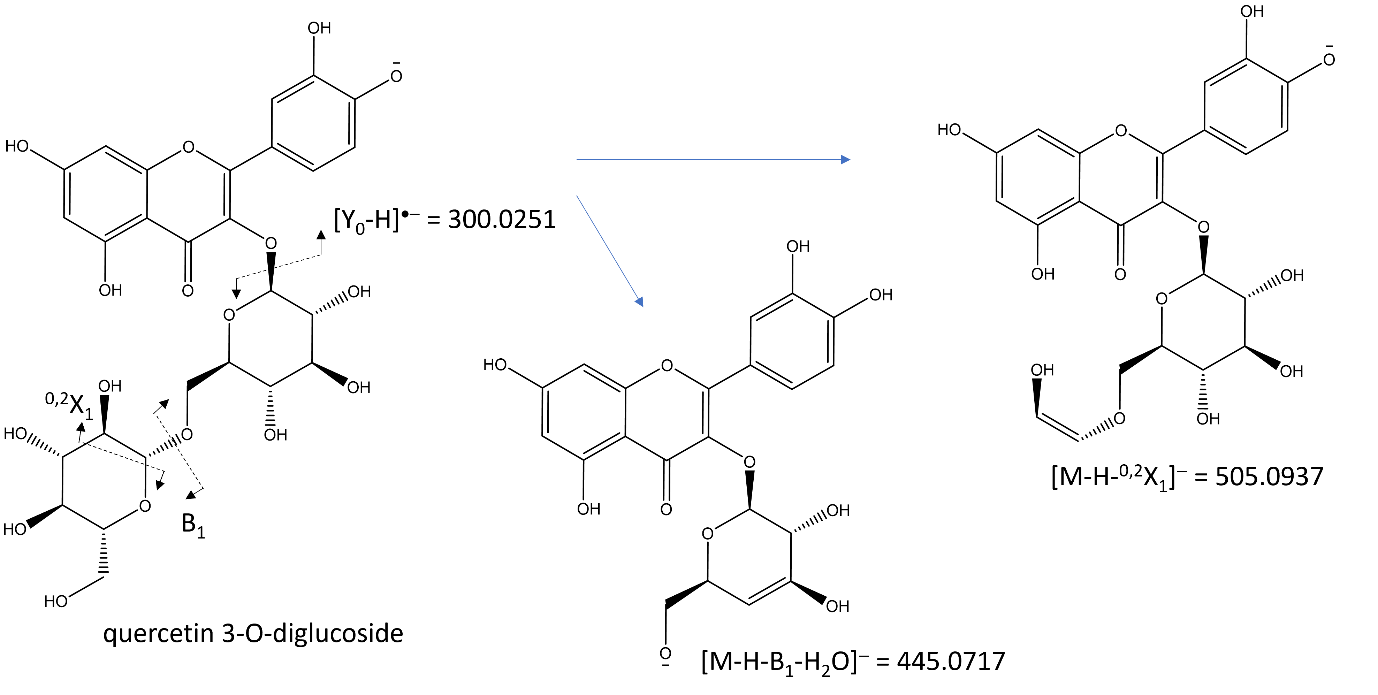


**Fig. S3.** The proposed fragmentation of quercetin 3-*O*-diglucoside, *m/z* 625.1444. The fragments ions of flavonoid glycosides were identified based on the fragment nomenclature system (Vukics and Guttman, 2010).


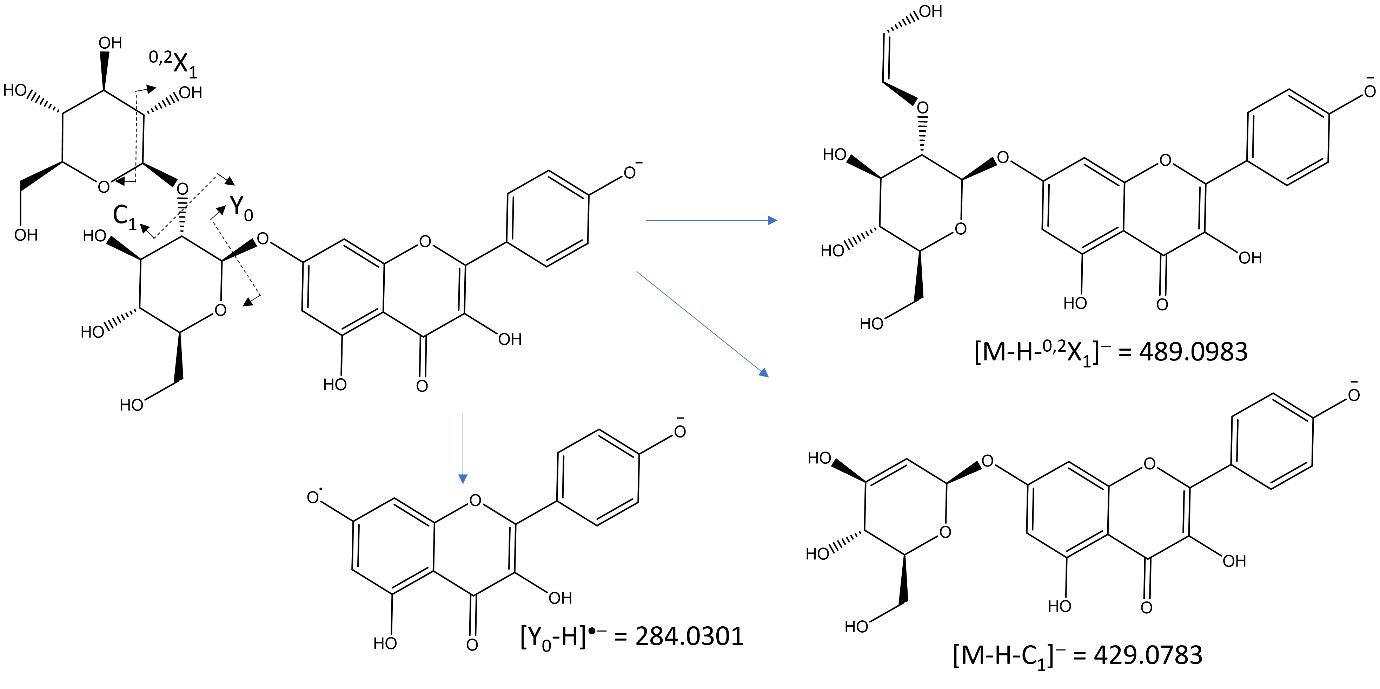


**Fig. S4.** The proposed fragmentation of kaempferol-7-sophoroside, *m/z* 609.1500. The fragments ions of flavonoid glycosides were identified based on the fragment nomenclature system (Vukics and Guttman, 2010).
